# Single immunization with genetically attenuated *Pf∆mei2* (GA2) parasites by mosquito bite in controlled human malaria infection: a placebo-controlled randomized trial

**DOI:** 10.1038/s41591-024-03347-2

**Published:** 2025-01-03

**Authors:** Geert V. T. Roozen, Roos van Schuijlenburg, Annefleur D. O. Hensen, Jan Pieter R. Koopman, Olivia A. C. Lamers, Fiona J. A. Geurten, Jeroen C. Sijtsma, Els Baalbergen, Jacqueline J. Janse, Séverine Chevalley-Maurel, Chanel M. Naar, Sascha Bezemer, Hans Kroeze, Huybert J. F. van de Stadt, Bram de Visser, Pauline Meij, Mara S. Tihaya, Emil Colstrup, Eva Iliopoulou, Helena M. de Bes-Roeleveld, Els Wessels, M. Y. Eileen C. van der Stoep, Chris J. Janse, Rajagopal Murugan, Blandine M. D. Franke-Fayard, Meta Roestenberg

**Affiliations:** 1 Leiden University Center for Infectious Diseases, Leiden University Medical Center, Leiden, The Netherlands; 2 Medical Technology and Prototyping, Leiden University Medical Center, Leiden, The Netherlands; 3 Center for Cell and Gene Therapy, Leiden University Medical Center, Leiden, The Netherlands; 4 Clinical Pharmacy and Toxicology, Leiden University Medical Center, Leiden, The Netherlands

**Keywords:** Malaria, Live attenuated vaccines

## Abstract

Malaria vaccines consisting of metabolically active *Plasmodium falciparum* (*Pf*) sporozoites can offer improved protection compared with currently deployed subunit vaccines. In a previous study, we demonstrated the superior protective efficacy of a three-dose regimen of late-arresting genetically attenuated parasites administered by mosquito bite (GA2-MB) compared with early-arresting counterparts (GA1-MB) against a homologous controlled human malaria infection. Encouraged by these results, we explored the potency of a single GA2-MB immunization in a placebo-controlled randomized trial. Primary outcomes were safety and tolerability, time-to-parasitemia and protective efficacy. Humoral and cellular immunological results were considered secondary outcomes. Here we report the safe administration of GA2-MB with no breakthrough malaria and sterile protection in nine of ten participants at 6 weeks after a single immunization with 50 GA2-infected mosquitoes, compared with none of five mock-immunized participants, against a homologous controlled human malaria infection. Immunization increased circulating *Pf*-specific polyfunctional effector memory CD4^+^ T cells coexpressing tumor necrosis factor and interleukin-2. This unprecedented 90% protective efficacy after a single low-dose immunization holds great promise for the potency of GA2 immunization. Future studies should demonstrate whether GA2 is similarly efficacious in pre-exposed populations and whether the favorable safety profile reported here holds up in larger groups. ClinicalTrials.gov registration: NCT05468606.

## Abstract (web summary)

In a small randomized controlled clinical trial, a single immunization for malaria using mosquitoes infected with attenuated parasites showed unprecedented 90% protective efficacy and did not lead to breakthrough disease.

## Main

Each year more than 600,000 people die from malaria, mainly children under the age of five^1^, making it the fifth leading cause of child mortality worldwide^2^. Although the widespread deployment of both RTS,S and R21 subunit vaccines in regions with moderate-to-high transmission marks tremendous progress in reducing malaria-related morbidity and mortality^3,4^, their limited efficacy and the need for boosters to sustain protection call for ongoing effort into the development of improved vaccines with increased potency to achieve high-level durable protection that can ultimately break transmission^5^. Immunization strategies based on the use of whole *Plasmodium falciparum* (*Pf*) sporozoites have the potential to provide this much sought after high-level protection, particularly late liver stage-arresting parasites that are attenuated genetically through the knockout of genes crucial for the development of blood-stage disease^6–9^.

Previously, we demonstrated that genetically attenuated *Pf* sporozoites can be safely administered to humans by injection and mosquito bite^10,11^. Through targeted gene deletion, we created two different parasite lines based on the *Pf* NF54 strain: early-arresting *Pf∆b9/∆slarp* (GA1)^12^ and late-arresting *Pf∆mei2* (GA2)^8^. We demonstrated with three immunizations through mosquito bites that parasites arresting development late in the liver at day 6 post-infection are much more potent in inducing protection than early-arresting counterparts (89% versus 13% protection in a controlled human malaria infection (CHMI))^11^. The high-level protection was accompanied by potent circulating cellular memory responses, potentially against late liver-stage antigens^11^.

In previous studies involving immunization with sporozoites under chemoprophylaxis, detailed parasite detection by quantitative polymerase chain reaction analysis for *Pf* (*Pf*qPCR) during immunization regimens indicated that 10–90% of previously malaria-naive participants become parasitemic after the second immunization^13–18^. This result suggests that single immunization with parasites that reach the late liver stage can provide varying levels of immunity. We hypothesized that GA2 might also have that potential and we decided to assess the efficacy of a single GA2-immunization regimen against a homologous CHMI.

## Results

**Fig. 1:**
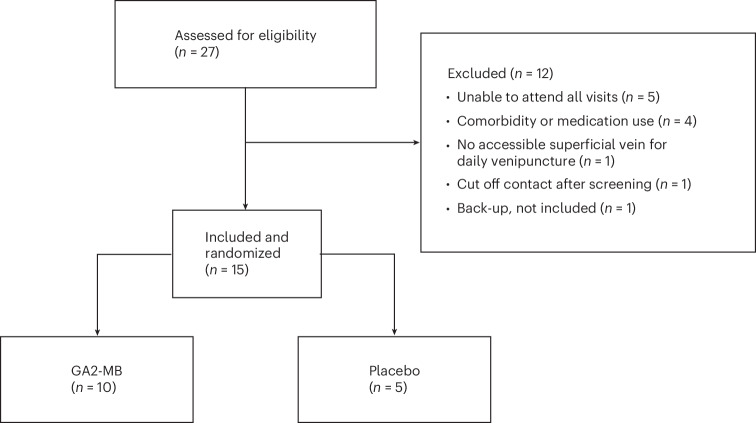
CONSORT diagram on recruitment and inclusions. Between 13 February 2023 and 17 March 2023, 27 persons were screened, of whom 15 were included, randomized, (mock-)immunized and infected with wild-type malaria parasites in a CHMI.

**Fig. 2:**
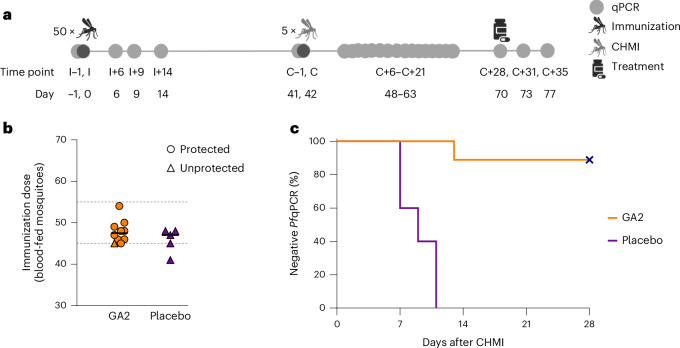
Study design, immunization dose and protection against CHMI. **a**, Schematic overview of study design. **b**, Number of blood-fed mosquitoes per participant at immunization. Black horizontal lines represent the median. The dashed horizontal line represents the target dose (45–55 blood-fed mosquitoes). **c**, Kaplan–Meier curve of percentage of participants that had a negative *Pf*qPCR (<100 parasites per ml) in peripheral blood after CHMI. X, censored; log-rank test, *P* < 0.0001.

**Extended Data Fig. 1:**
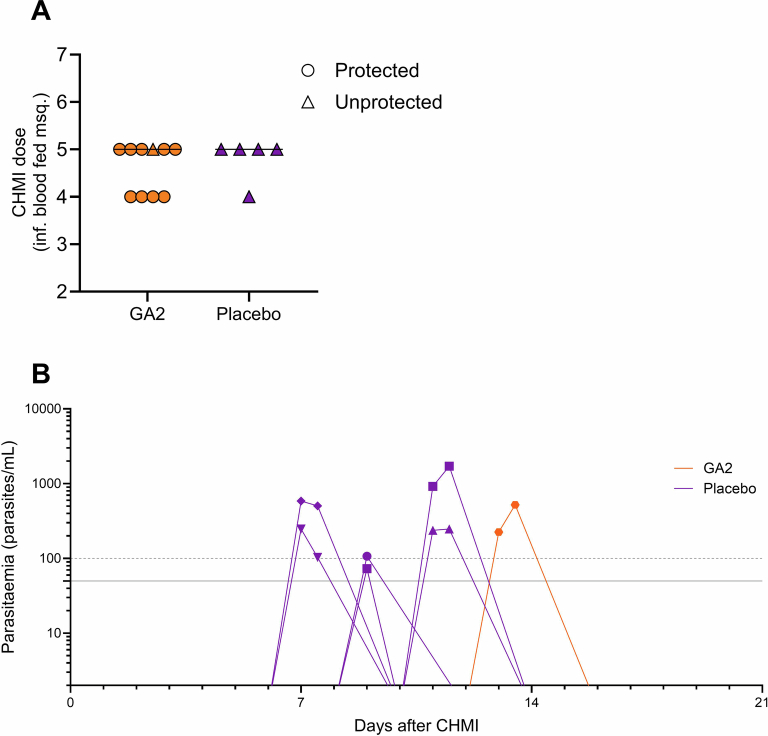
CHMI dose and time-to-parasitaemia. **A**. Number of infected blood fed mosquitoes per participant at the controlled human malaria infection (CHMI). A black horizontal line represents the median. **B**. Development of parasitaemia per day after CHMI. Lines with different symbols represent different participants. The horizontal dotted grey line represents the cut-off for parasitaemia (100 parasites/mL blood) at which treatment is given. The horizontal continuous grey line represents the lowest limit of detection of the assay (50 parasites/mL blood).

We enrolled 15 participants in a randomized double-blind placebo-controlled trial. Details on recruitment and participant characteristics can be found in Fig. 1 and Table 1. Participants were exposed to the bites of 50 (±5) GA2-infected or uninfected *Anopheles stephensi* mosquitoes (GA2-MB or placebo, respectively) on 12 April 2023 (Fig. 2a). For one participant in the placebo group, the target dose of 45–55 blood-fed mosquitoes was not reached at immunization (Fig. 2b). Six weeks later, all participants underwent CHMI through the bites of five mosquitoes infected with unattenuated homologous wild-type *Pf* parasites. After CHMI, blood feedings were confirmed in either five infected mosquitoes (6 of 10 GA2-MB participants and 4 of 5 in the placebo group) or four infected mosquitoes (4 of 10 GA2-MB participants and 1 of 5 in the placebo group) (Extended Data Fig. 1a).

**Table 1:** Baseline characteristics of participants at screening

Characteristics	GA2-MB (*n* = 10)	Placebo (*n* = 5)	Total (*n* = 15)
Age			
Mean (s.d.)	24 (3)	24 (5)	24 (4)
Median (range)	23 (21–30)	25 (19–31)	23 (19–31)
Sex			
Male (%)	4 (40.0)	3 (60.0)	7 (46.7)
Female (%)	6 (60.0)	2 (40.0)	8 (53.3)
BMI			
Mean (s.d.)	23.9 (3.3)	22.7 (2.1)	23.5 (2.9)
Median (range)	23.8 (19.4-28.6)	23.7 (20.3-25.0)	23.7 (19.4-28.6)

BMI, body mass index; s.d., standard deviation.

Study visits were held on the day before immunization, on days 6, 9 and 14 post-immunization, on the day before CHMI, daily from day 6 to day 21 post-CHMI, and on days 28, 31 and 35 post-CHMI. During these visits, adverse events (AEs) were collected, safety was assessed and a highly sensitive *Pf*qPCR analysis in whole blood was performed. The lowest limit of detection for the *Pf*qPCR was 50 parasites per ml of blood. Escape treatment (3-day regimen of atovaquone–proguanil) was provided at a concentration of >100 parasites per ml or at day 28 after CHMI.

### Primary outcomes

Single immunization with GA2-MB was safe and well tolerated with no study-related serious AEs or breakthrough malaria. Neither were parasite concentrations ≥50 parasites per ml detected in any blood sample after immunization. The mosquito bites led to a severe itch in one participant and moderate swelling and mild blistering in another participant, for which topical corticosteroids were prescribed. All other AEs after immunization were mild to moderate. Further details on safety and tolerability can be found in Extended Data Table 1.

We found that 9 of 10 (90%) participants in the GA2-MB group were fully protected against *Pf* malaria and remained *Pf*qPCR-negative until day 28 post-CHMI (Fig. 2c). By contrast, all participants in the placebo group became parasitemic (log-rank test *P* < 0.0001). Although the median time-to-parasitemia in the placebo group was 9 days (range 7–11 days), detection of parasitemia in the one unprotected GA2-MB participant was considerably delayed to day 13 (Extended Data Fig. 1b). When assessing protection solely in the participants that received a dose of five infected blood-fed mosquitoes at CHMI (not a prespecified analysis in our protocol), we found a protective efficacy of 83% (5 of 6 GA2-MB participants protected versus 0 of 4 placebo participants).

### Secondary outcomes

**Fig. 3:**
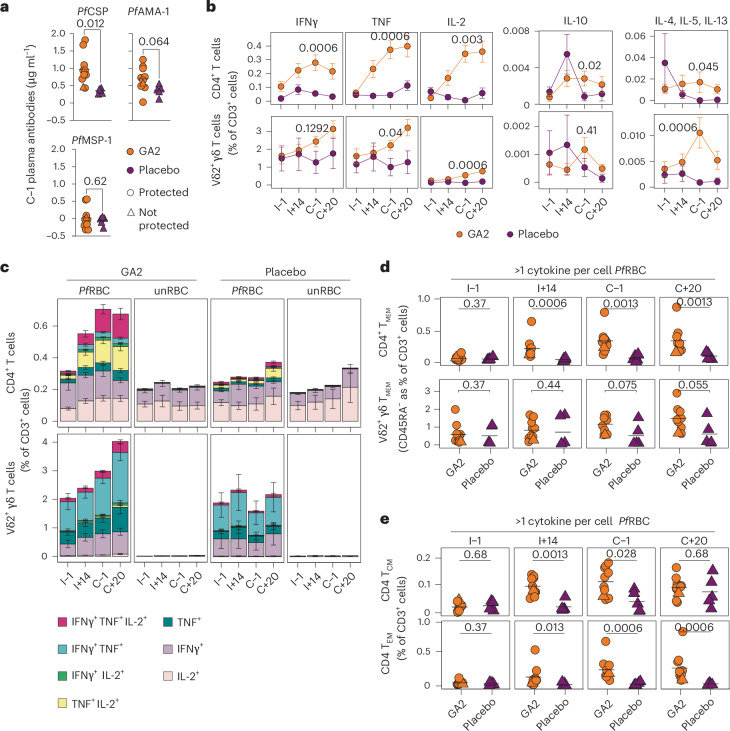
Prominent polyfunctional CD4^+^ memory T cell response in GA2 single-immunized participants. **a**, Plasma antibody levels against the indicated *Pf* antigens on the day before CHMI (C−1). Values are log_10_ transformed. **b**, Frequency of CD4^+^ and Vδ2^+^ γδ T cells expressing the indicated cytokines upon stimulation with *Pf* RBC corrected for unRBC stimulation. **c**, Frequency of CD4^+^ and Vδ2^+^ γδ T cells expressing single or more than one of the indicated cytokines per cell upon *Pf* RBC and unRBC stimulation. **d**, Frequency of CD45RA^−^ CD4^+^ (upper) and Vδ2^+^ γδ (lower) memory T cells (T_MEM_) among polyfunctional cells at the indicated time points upon *Pf* RBC stimulation. **e**, Frequency of central (T_CM_) (upper) and effector (T_EM_) (lower) memory cells among polyfunctional CD4^+^ T cells. Filled circles and triangles indicate the data from individual participants and the horizontal black line indicates the arithmetic mean (**a**, **d**, **e**). Filled circles and error bars indicate the arithmetic mean and s.e.m., respectively (**b**). Bar charts represent arithmetic means and error bars represent s.e.m. (**c**). Two-tailed Mann–Whitney test (**a**, **b** (at C−1), **d**, **e**). *Pf*AMA-1, *Pf* apical membrane antigen-1 ; *Pf*CSP, *Pf* circumsporozoite protein; *Pf* MSP-1, *Pf* merozoite surface protein-1.

**Extended Data Fig. 2:**
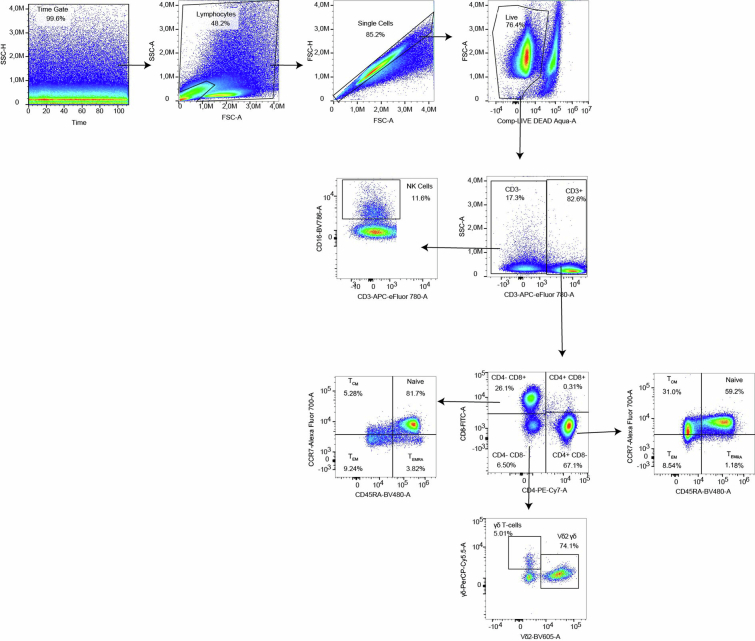
Representative gating strategy to define T cell subsets. Live single cells were defined as NK cells (CD3^−^CD56^+^), CD4 or CD8 effector (CCR7^−^CD45RA^−^) and central (CCR7^+^CD45RA^−^) memory T cells and Vδ2^+^ or Vδ2^−^ γδ T cells, based on the surface marker expression.

**Extended Data Fig. 3:**
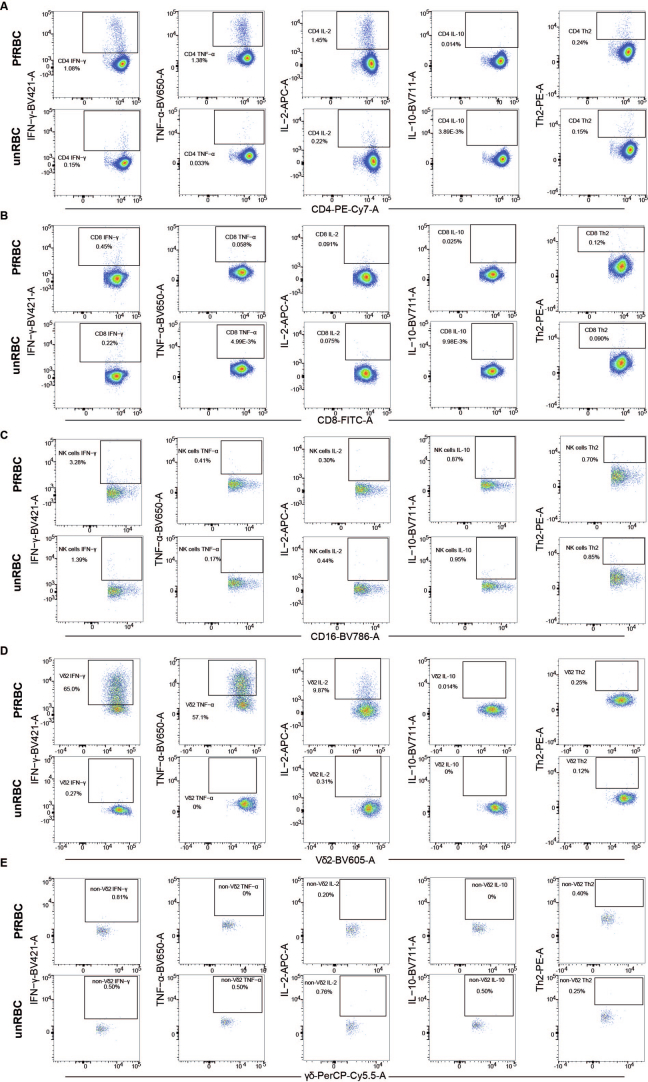
Representative cytokine expression gating strategy. Gates defining the indicated cytokine expressing CD4^+^ (**A**), CD8^+^ (**B**), NK (**C**), Vδ2^+^ γδ (**D**) and Vδ2^−^ γδ (**E**) T cells upon stimulation with *Pf* infected (*Pf* RBC; top) and uninfected RBC (unRBC).

**Extended Data Fig. 4:**
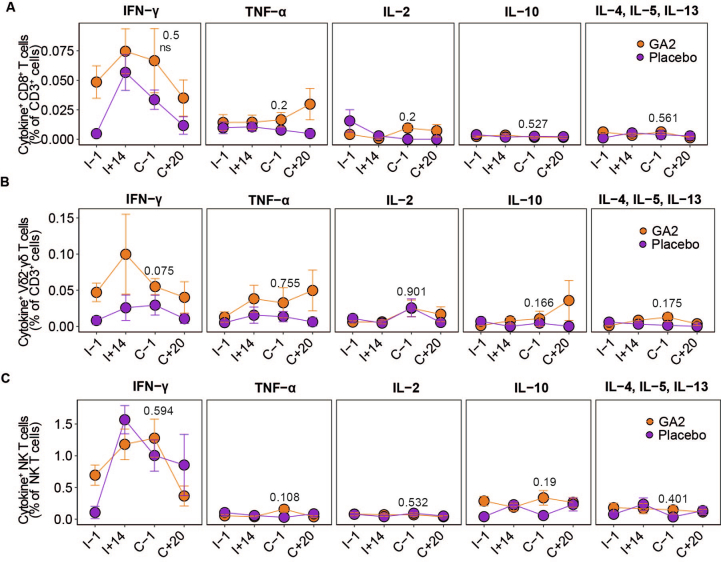
Cytokine expression in T cell subsets. CD8^+^ (**A**), Vδ2^-^ γδ (**B**) and, NK (**C**) T cells expressing the indicated cytokines upon stimulation with *Pf* infected RBC (*Pf* RBC) reference corrected for uninfected RBC (unRBC) stimulation. Data corresponding to GA2-MB and placebo groups are indicated in orange and purple, respectively (**A–C**). Filled circles and error bars indicate arithmetic mean and standard error mean, respectively. Two tailed Mann-Whitney test (at C-1).

**Extended Data Fig. 5:**
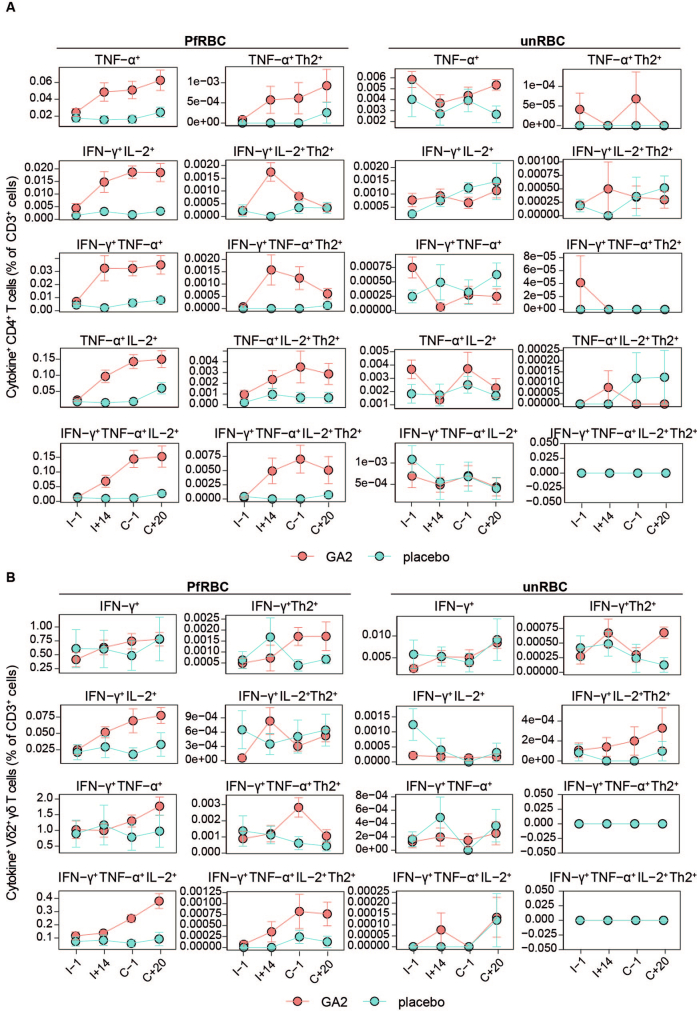
Mono and polyfunctional T cells moderately co-expressing Th2 cytokines. Frequency of CD4^+^ (**A**) and Vδ2^+^ γδ (**B**) T cells expressing the indicated cytokines in combination with or without type-2 cytokines (Th2: IL-4, IL-5 and IL-13) among GA2-MB and mock-immunised participants upon *Pf* RBC (left) and unRBC (right) stimulation. Filled circles and error bars indicate arithmetic mean and standard error of mean of both intervention groups, respectively.

**Extended Data Fig. 6:**
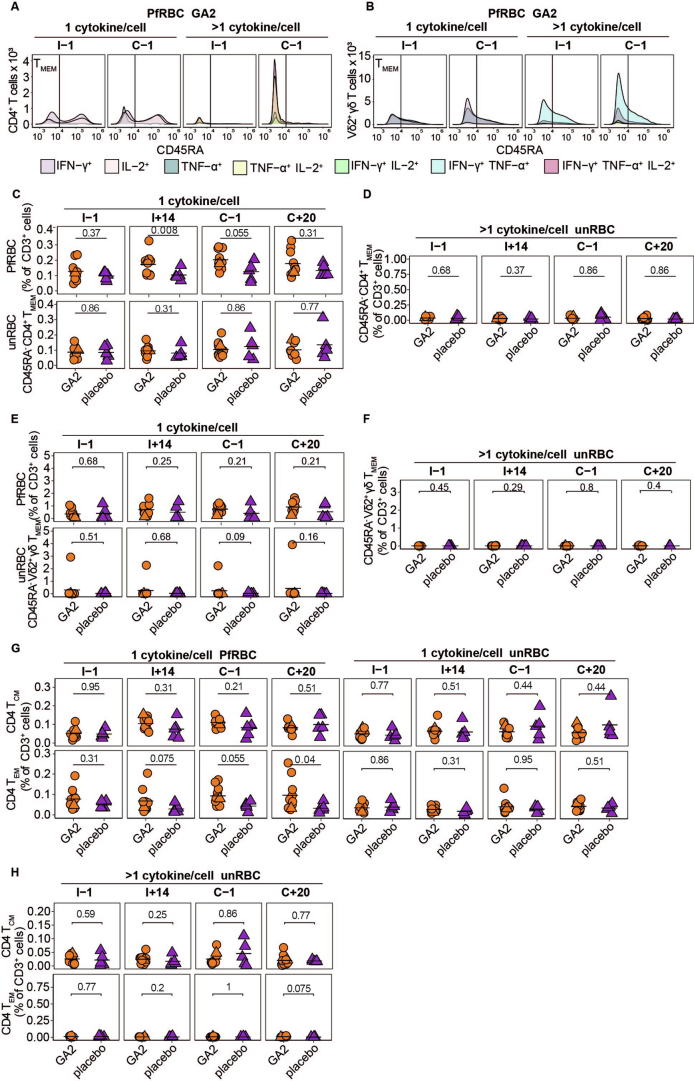
Memory status of T cell subsets. **A,B**. CD45RA expression in CD4^+^ (**A**) and Vδ2^+^ γδ (**B**) T cells among cytokine expressing cells at baseline (I-1) and C-1 upon *Pf* RBC stimulation. **C-F**. Frequency of CD45RA^−^ mono^-^ (**C**,**E**) and polyfunctional (**D**,**F**) CD4^+^ (**C**,**D**) and Vδ2^+^ γδ (**E**,**F**) memory T cells (T_MEM_) upon *Pf* RBC and unRBC stimulation. **G,H**. Frequency of central (T_CM_; top) and effector (T_EM_; bottom) memory cells among monofunctional and polyfunctional in CD4^+^ (**G**) and Vδ2^+^ γδ (**H**) T cells. Each data point represent individual participant and data corresponding to GA2-MB and placebo groups are indicated in orange and purple, respectively (**C-H**). Horizontal lines indicate arithmetic mean (**C-H**). Two tailed Mann-Whitney test.

We assessed antibody responses 1 day before CHMI (C−1) and detected significantly higher levels of antibodies targeting *Pf* circumsporozoite protein, but not the key late liver-stage and blood-stage antigens *Pf* apical membrane antigen-1 and *Pf* merozoite surface protein-1 in GA2-MB participants compared with placebo (Fig. 3a). *Pf*-specific cellular immunity in GA2-MB participants assessed by stimulation of peripheral blood mononuclear cells with *Pf*-infected red blood cells (*Pf* RBC), a surrogate for late liver-stage antigens, and uninfected RBCs (unRBCs) showed a strong type-1 proinflammatory (interferon-γ (IFNγ), tumor necrosis factor (TNF) and interleukin-2 (IL-2)), and a moderate type-2 anti-inflammatory (IL-4, IL-5 and IL-13) and regulatory (IL-10) profile in CD4^+^ and Vδ2^+^ γδ T cells, but not in CD8^+^, Vδ2^–^ γδ and natural killer T cells (Fig. 3b and Extended Data Figs. 2–4). GA2-MB elicited higher frequencies of polyfunctional CD4^+^ and Vδ2^+^ γδ T cells expressing more than one type-1 cytokine, in comparison with placebo (Fig. 3c). Whereas CD4^+^ T cells preferentially coexpressed TNF and IL-2 with or without IFNγ, Vδ2^+^ γδ T cells coexpressed high levels of IFNγ and TNF with or without IL-2. We observed a relatively minor proportion of type-1 polyfunctional CD4^+^ and Vδ2^+^ γδ T cells coexpressing type-2 cytokines (Extended Data Fig. 5). Polyfunctional CD4^+^ T cells, but not Vδ2^+^ γδ T cells, were enriched among memory T cells (CD3^+^CD45RA^−^), indicating the capacity of single GA2-MB immunization to form *Pf*-specific cellular memory (Fig. 3d and Extended Data Fig. 6a–f). These GA2-induced memory T cells preferentially acquired effector memory phenotype as early as 2 weeks post-immunization and remained high during the post-CHMI time point, whereas central memory T cells were induced at much lower frequency (Fig. 3e and Extended Data Fig. 6g,h).

## Discussion

In this study, we demonstrated the capacity of a single immunization with 50 GA2-infected mosquitoes to protect 90% of malaria-naive individuals against a homologous CHMI. Until now, malaria vaccines have always been tested in regimens of two or more immunizations, but single immunization has important potential advantages over multiple immunizations with regards to implementation in endemic settings as well as for travelers’ vaccinations. Although high-level protective efficacy after immunization with sporozoites has been observed previously^11,13–19^, never has protective efficacy been demonstrated in a CHMI after one immunization. Surprisingly, this high level of protective efficacy seems to be similar to three immunizations with GA2-infected mosquito bites^11^, suggesting that the boosting effect of additional immunizations is limited. Further research into the dynamics of immune components in a larger cohort of participants is needed to understand changes on an individual basis and to evaluate how these changes relate to protection. In addition, the longevity of the immune response and protective efficacy after both single and triple GA2-MB immunization needs to be further evaluated and compared.

Blood-stage breakthrough infections have been observed after immunizing mice with *Plasmodium yoelli ∆mei2* (ref. ^20^). When taking the current and our previous GA2-MB study^11^ together, a total of 50 participants have now been exposed to GA2-MB: 15 participants to 15 GA2-MB and 35 participants to 50 GA2-MB (nine of whom underwent three exposures). None of these participants have developed breakthrough malaria. This is in line with results in mice with humanized livers that were exposed to *Pf∆mei2* and did not develop breakthrough blood infections either^8^. Future studies should demonstrate whether this favorable safety profile of GA2 holds in larger populations or whether genetically attenuated parasites with more gene knockouts in addition to *mei2* are warranted to eliminate the risk of breakthrough infections after immunizations^21^.

Because of the lack of knowledge on immunogenic late liver-stage antigens and the technical limitations of generating large numbers of infected hepatocytes suitable for in vitro stimulation, *Pf* RBCs were used as a surrogate antigenic source in this study, similar to previously published clinical studies^10,17,19^. Furthermore, the preferential liver resident capacity of *Pf*-specific CD8^+^ T cells may have thwarted our attempts to detect them in peripheral blood samples. In previous sporozoite immunization studies, polyfunctional CD4^+^ and Vδ2^+^ γδ T cells expressing proinflammatory cytokines, particularly IFNγ, were associated with protection^11,13,17,19^. However, after single GA2-MB immunization we find the production of proinflammatory cytokines other than IFNγ (notably TNF and IL-2) by CD4^+^ T cells to be more pronounced, in addition to an increase in effector rather than central memory phenotypes, which differentiate early after immunization and persist throughout the CHMI follow-up period.

A limitation of our study is the small sample size of healthy malaria-naive participants who do not adequately represent the target population for malaria vaccines in endemic areas. In addition, administration of GA2 through mosquito bites is not a feasible method for large-scale immunization campaigns. To translate the high-level protective efficacy of GA2-MB to an amenable way of vaccine administration through parenteral immunization, future studies need to assess whether aseptically purified, vialed and cryopreserved sporozoites with the *mei2* deletion are as safe and as efficacious as GA2-MB in this study and similarly potent in malaria endemic areas.

Nonetheless, our finding that a single immunization with GA2-MB can induce high-level protection against a homologous CHMI provides strong support for the further clinical development of potentially highly potent next generation single-immunization malaria vaccines based on late-arresting genetically attenuated sporozoites.

## Methods

### Study design and recruitment

A randomized, double-blind, placebo-controlled trial with a CHMI was conducted from February to November 2023 at Leiden University Medical Center, Leiden, the Netherlands. Fifteen malaria-naive participants aged 15–30 years were included after a health assessment including medical history, physical examination, a general laboratory evaluation including hematology and biochemistry assessment, a drugs test to exclude cocaine and amphetamine use and electrocardiography. Female participants were counseled to use adequate contraception throughout the study and were tested for pregnancy with a serum beta-human chorionic gonadotropin test on both the day before immunization (I−1) and the day before CHMI (C−1). All participants provided written informed consent.

Ten participants were immunized with the bites of 45–55 GA2-infected mosquitoes and five participants received a mock-immunization with uninfected mosquitoes as a placebo. Six weeks after immunization, all 15 participants underwent a homologous CHMI with the bites of five wild-type *Pf* 3D7-infected mosquitoes. From day 6 to day 21 after CHMI (C+6 to C+21), participants were closely followed with daily ambulatory visits for the collection of AEs, safety assessment, blood sampling and highly sensitive *Pf*qPCR analysis in whole blood as previously described (the lowest limit of detection was 50 parasites per ml)^22^. Participants were treated with a 3-day regimen of atovaquone–proguanil when they exhibited parasitemia (*Pf*qPCR > 100 parasites per ml) or at day 28 after CHMI (C+28). AEs were recorded by participants in a diary. AEs were graded in four categories (mild, moderate, severe and serious) that were prespecified per protocol. Both participants and investigators were blinded to intervention. Mosquito cages were prepared by technicians independent from the clinical investigators. Randomization was carried out by an independent member of the study team. Safety, time-to-parasitemia and protective efficacy were the primary outcomes. Secondary study outcomes were humoral and cellular immunology results. Data capture was done using an electronic case report form (Castor CDMS v.2023.1.x.x).

The trial protocol was approved by the Dutch Central Committee for Research involving Human Subjects (CCMO, file number NL82130.000.22) and registered at ClinicalTrials.gov (NCT05468606) and EudraCT (2022-002646-40).

### Parasite culturing, mosquito rearing and exposures

The characterization of GA2 and its generation from *Pf* NF54, its genetic backbone, have been described previously^8^. The wild-type parasite used for the CHMI (*Pf* 3D7) is a clone of the *Pf* NF54 parasite strain. Parasites were cultured in standard conditions using semi-automated shaker culture systems^23^ and subsequently fed to female *Anopheles stephensi* mosquitoes by standard membrane feeding^24^. Mosquitoes were reared and infected following standard procedures at the insectary of Leiden University Medical Center following established methods^24^. Production of the parasites and mosquitoes underwent strict quality control before release by a qualified person. Fourteen days after feeding the parasites to the mosquitoes, a sample of 20 mosquitoes was taken from every mosquito batch (consisting of 200–500 mosquitoes) to assess sporozoite yield in the mosquito salivary glands. Only batches that had an average yield of at least 1,000 sporozoites per mosquito were used for exposure to participants. For the immunization, the average yield of the batches was 34,000 and 67,000 sporozoites per mosquito and for the challenge the yield of the batches ranged from 11,300 to 35,300 sporozoites per mosquito.

Exposure of mosquitoes to participants was done using small cages with mesh-covered openings that were applied for 15 min to the deltoid region (immunization) or inner lower arm (CHMI). After exposure, mosquitoes were dissected to confirm feeding. In addition, after CHMI exposures, salivary glands were dissected and microscopically assessed for the presence of sporozoites. At immunization, exposures were repeated until the target dose of 45–55 mosquitoes was reached or up to a maximum of three times. For CHMI, the procedure was repeated until five infected mosquitoes had taken a blood meal or up to a maximum of four exposures.

### *Pf* antigen-binding antibody measurements in ELISA

ELISA were performed as described previously^11^. In brief, half-area 96-well high-binding plates were coated overnight at 4 °C with 1 µg ml^−1^ of antigen at 25 µl per well in 0.1 M sodium carbonate buffer (pH 9.6). Upon blocking with 5% skim milk in phosphate-buffered saline for 2 h, serially diluted plasma samples (starting dilution of 1:500 serially diluted in eight steps each by 1:2.5) were incubated for 2 h. Bound antibodies were detected with 450 nm absorbance using goat anti-human immunoglobulin G conjugated with horseradish peroxidase and 3,3′,5,5′-tetramethylbenzidine substrate development stopped with 10% sulfuric acid. A standard curve developed using polyclonal immunoglobulin G of a known concentration was used for normalization. Measures from at least two independent experiments with a coefficient of variance below 30% were considered for analysis.

### T cell response measurement using flow cytometry

Cellular response using *Pf* RBC stimulation was performed as described previously^5^. In brief, peripheral blood mononuclear cells were stimulated with RBCs from a healthy blood donor, either as unRBC or *Pf* RBC, for 24 h during which 10 µg ml^−1^ Brefeldin A (Sigma) was added at 4 h post stimulation. Cells were stained with a panel of antibodies (Extended Data Table 2) to identify T cell subsets (CD56, γδ Vδ2 T cell receptor, CCR7, CD3, CD4, CD8, CD25 and CD11c), cytokine expression (IFNγ, TNF, IL-10, IL-2, IL-4, IL-5, IL-13) and phenotype (CD45RA and CCR7). For fixation and intracellular staining an Intracellular Fixation & Permeabilization Buffer Set (Invitrogen) was used. To stain dead cells Aqua Live/Dead dye (Invitrogen) was used. Cells were acquired on the three-laser spectral analyzer Aurora (configuration 16V-14B-8R) and analyzed using FlowJo v.10.8.2 as described in Extended Data Figs. 2 and 3. The frequency of *Pf*-specific cytokine-positive cells in *Pf* RBC-stimulated samples after subtraction of the same gate on the same sample stimulated with unRBC is reported for analysis. Frequencies of cytokine-positive CD4^+^ and γδ^+^ Vδ2 cells were calculated as a percentage of CD3^+^ cells by using the frequencies of total CD4^+^ and γδ^+^ Vδ2 cells, respectively.

### Statistical analysis

Baseline characteristics of participants are reported as both means with standard deviations and medians with range for continuous variables and as frequencies with percentages for categorical variables. The incidence of AEs is reported as frequencies with percentages (risk). Time-to-parasitemia is reported as a Kaplan–Meier graph and the difference between groups is evaluated using a log-rank test. Antibody concentrations are reported in µg ml^−1^ and the frequency of responding cells as a percentage of the indicated population, both as arithmetic means with standard error of means. Antibody concentrations and cell populations are compared between groups with a two-tailed Mann–Whitney test.

Figures were produced in GraphPad Prism (v.9.3.1) and RStudio (v.4.2.1).

### Reporting summary

Further information on research design is available in the Nature Portfolio Reporting Summary linked to this article.

## Online content

Any methods, additional references, Nature Portfolio reporting summaries, source data, extended data, supplementary information, acknowledgements, peer review information; details of author contributions and competing interests; and statements of data and code availability are available at 10.1038/s41591-024-03347-2.

## Supplementary information

Reporting Summary

## Figures and Tables

**Extended Data Table 1:**
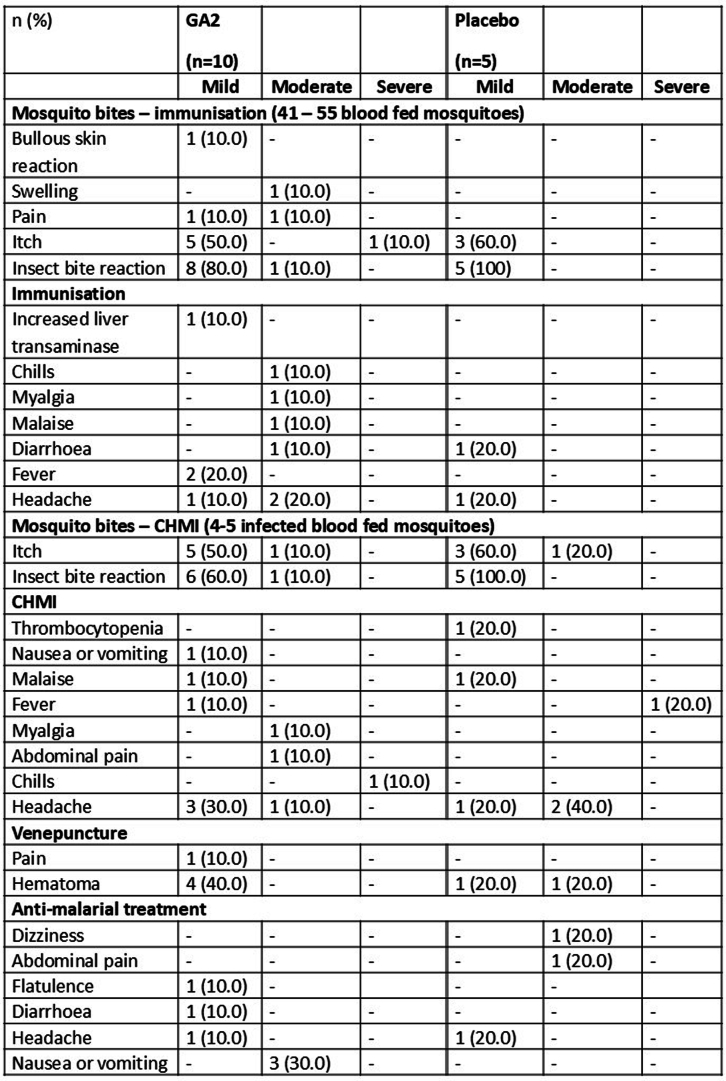
Risk of study related adverse events

CHMI = controlled human malaria infection.

**Extended Data Table 2:**
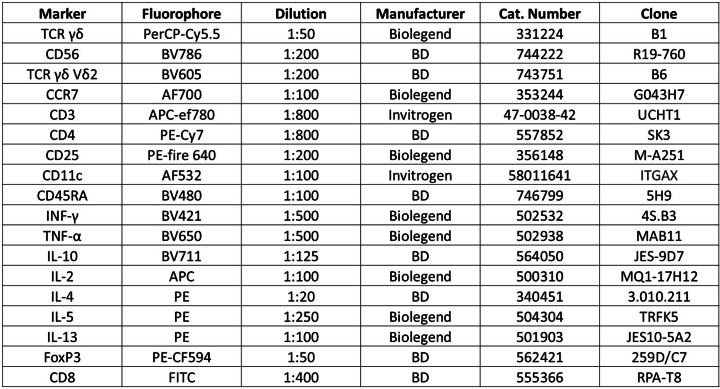
Staining antibody panel used in multi-parameter spectro-flow cytometry

## Data Availability

For the full study protocol or raw data, contact m.roestenberg@lumc.nl. Deidentified participant data can be shared. External data requests will be answered within 1 month.
