# Supplementary material for: Single immunization with genetically attenuated Pf∆mei2 (GA2) parasites by mosquito bite in controlled human malaria infection: a placebo-controlled randomized trial
Source: Nat Med. 2025 Jan 3;31(1):218–22. doi: 10.1038/s41591-024-03347-2 (PMC11750698; doi:10.1038/s41591-024-03347-2)
Supplement: Supplementary file 1 — Reporting Summary [file 41591_2024_3347_MOESM1_ESM.pdf]

nature portfolio

Corresponding author(s): Meta Roestenberg

Last updated by author(s): 01-OCT-2024

Reporting Summary

Nature Portfolio wishes to improve the reproducibility of the work that we publish. This form provides structure for consistency and transparency in reporting. For further information on Nature Portfolio policies, see our Editorial Policies and the Editorial Policy Checklist.

Statistics

For all statistical analyses, confirm that the following items are present in the figure legend, table legend, main text, or Methods section.

n/a	Confirmed	
☐	☒	The exact sample size (*n*) for each experimental group/condition, given as a discrete number and unit of measurement
☐	☒	A statement on whether measurements were taken from distinct samples or whether the same sample was measured repeatedly
☐	☒	The statistical test(s) used AND whether they are one- or two-sided<br>*Only common tests should be described solely by name; describe more complex techniques in the Methods section.*
☒	☐	A description of all covariates tested
☒	☐	A description of any assumptions or corrections, such as tests of normality and adjustment for multiple comparisons
☐	☒	A full description of the statistical parameters including central tendency (e.g. means) or other basic estimates (e.g. regression coefficient) AND variation (e.g. standard deviation) or associated estimates of uncertainty (e.g. confidence intervals)
☐	☒	For null hypothesis testing, the test statistic (e.g. *F*, *t*, *r*) with confidence intervals, effect sizes, degrees of freedom and *P* value noted<br>*Give P values as exact values whenever suitable.*
☒	☐	For Bayesian analysis, information on the choice of priors and Markov chain Monte Carlo settings
☒	☐	For hierarchical and complex designs, identification of the appropriate level for tests and full reporting of outcomes
☒	☐	Estimates of effect sizes (e.g. Cohen's *d*, Pearson's *r*), indicating how they were calculated

Our web collection on statistics for biologists contains articles on many of the points above.

Software and code

Policy information about availability of computer code

Data collection	Data has been collected using eCRF software from Castor CDMS (version 2023.1.x.x).
Data analysis	Figures were produced in GraphPad Prism (version 9.3.1) and RStudio (version 4.2.1).

For manuscripts utilizing custom algorithms or software that are central to the research but not yet described in published literature, software must be made available to editors and reviewers. We strongly encourage code deposition in a community repository (e.g. GitHub). See the Nature Portfolio guidelines for submitting code & software for further information.

Data

Policy information about availability of data

All manuscripts must include a data availability statement. This statement should provide the following information, where applicable:

- Accession codes, unique identifiers, or web links for publicly available datasets
- A description of any restrictions on data availability
- For clinical datasets or third party data, please ensure that the statement adheres to our policy

For the full study protocol or raw data, contact m.roestenberg@lumc.nl. De-identified participant data can be shared. External data requests will be answered within one month.

Human research participants

Policy information about studies involving human research participants and Sex and Gender in Research.

Reporting on sex and gender	Biological sex is reported.
Population characteristics	Sex, age and BMI are reported in detail in Table 1. No relevant co-morbidities since only healthy participants were eligible.
Recruitment	Healthy Dutcht malaria-naïve adults were recruited from a participant datase, through social media and with posters in educational institutions. No reason to suspect relevant self-selection bias.
Ethics oversight	Ethical approval by Dutch national review board: Central Committee for Research Involving Human Subjects (CCMO).

Note that full information on the approval of the study protocol must also be provided in the manuscript.

Field-specific reporting

Please select the one below that is the best fit for your research. If you are not sure, read the appropriate sections before making your selection.

☒ Life sciences ☐ Behavioural & social sciences ☐ Ecological, evolutionary & environmental sciences

For a reference copy of the document with all sections, see nature.com/documents/nr-reporting-summary-flat.pdf

Life sciences study design

All studies must disclose on these points even when the disclosure is negative.

Sample size	The overall aim for malaria vaccine protective efficacy based on the WHO TPP is at least 70%. The GA2-MB group with 10 individuals has sufficient power (90% and an alpha of 5%) to determine this difference, when assuming a CHMI attack rate of 100%.
Data exclusions	12 participants excluded at screening. Details are reported in Figure 1.
Replication	(Mock-)immunisation and malaria challenge were performed once in every participant. For antibody assessment, measures from at least two independent experiments with a coefficient of variance below 30% were considered for analysis.
Randomization	Randomised 2:1 (GA2-MB : placebo)
Blinding	Doube-blind (participants and investigators)

Reporting for specific materials, systems and methods

We require information from authors about some types of materials, experimental systems and methods used in many studies. Here, indicate whether each material, system or method listed is relevant to your study. If you are not sure if a list item applies to your research, read the appropriate section before selecting a response.

Materials & experimental systems

n/a	Involved in the study	
☐	☒	Antibodies
☒	☐	Eukaryotic cell lines
☒	☐	Palaeontology and archaeology
☒	☐	Animals and other organisms
☐	☒	Clinical data
☒	☐	Dual use research of concern

Methods

n/a	Involved in the study	
☒	☐	ChIP-seq
☐	☒	Flow cytometry
☒	☐	MRI-based neuroimaging

Antibodies

Antibodies used	All use antibodies were purchased from commerical vendor as indicated in Extended Data Table 2.
Validation	Comercial antibodies were purchased with the valid vertificate of analysis. Details in the manufacturer's websites as referenced below:<br>https://www.biolegend.com/en-gb/products/percp-cyanine5-5-anti-human-tcr-gamma-delta-antibody-9237?GroupID=BLG5697

https://www.bdbiosciences.com/en-us/products/reagents/flow-cytometry-reagents/research-reagents/single-color-antibodies-ruo/bv786-mouse-anti-human-ncam-1-cd56.744222
https://www.bdbiosciences.com/en-dk/products/reagents/flow-cytometry-reagents/research-reagents/single-color-antibodies-ruo/bv605-mouse-anti-human-v-2-tcr.743751
https://www.biolegend.com/fr-ch/explore-new-products/alexa-fluor-700-anti-human-cd197-ccr7-antibody-13407?GroupID=BLG9610
https://www.thermofisher.com/antibody/product/CD3-Antibody-clone-UCHT1-Monoclonal/47-0038-42
https://www.bdbiosciences.com/en-nl/products/reagents/flow-cytometry-reagents/research-reagents/single-color-antibodies-ruo/pe-cy-7-mouse-anti-human-cd4.557852
https://www.biolegend.com/fr-fr/products/pe-fire-640-anti-human-cd25-antibody-20510
https://www.fishersci.ca/shop/products/cd11c-monoclonal-antibody-3-9-alexa-fluor-532-ebioscience-invitrogen/58011641
https://www.bdbiosciences.com/en-us/products/reagents/flow-cytometry-reagents/research-reagents/single-color-antibodies-ruo/bv480-mouse-anti-human-cd45ra.746799
https://www.biolegend.com/de-de/products/brilliant-violet-421-anti-human-ifn-gamma-antibody-7189
https://www.biolegend.com/nl-be/products/brilliant-violet-650-anti-human-tnf-alpha-antibody-7680
https://www.bdbiosciences.com/en-us/products/reagents/flow-cytometry-reagents/research-reagents/single-color-antibodies-ruo/BV711-Rat-Anti-Human-and-Viral-IL-10-.564050
https://www.biolegend.com/nl-be/products/apc-anti-human-il-2-antibody-1348
https://www.bdbiosciences.com/en-nl/products/reagents/flow-cytometry-reagents/clinical-discovery-research/single-color-antibodies-ruo-gmp/pe-mouse-anti-human-il-4.340451
https://www.biolegend.com/nl-nl/products/pe-anti-mouse-human-il-5-antibody-991
https://www.biolegend.com/de-at/products/pe-anti-human-il-13-antibody-940?GroupID=GROUP24
https://www.bdbiosciences.com/en-nl/products/reagents/flow-cytometry-reagents/research-reagents/single-color-antibodies-ruo/pe-cf594-mouse-anti-human-foxp3.562421
https://www.bdbiosciences.com/en-nl/products/reagents/flow-cytometry-reagents/research-reagents/single-color-antibodies-ruo/fitc-mouse-anti-human-cd8.555366

Clinical data

Policy information about clinical studies

All manuscripts should comply with the ICMJE guidelines for publication of clinical research and a completed CONSORT checklist must be included with all submissions.

Clinical trial registration	NCT05468606 and Eudra-CT 2022-002646-40
Study protocol	Version 3.0, 13-JAN-2023. The full trial protocol can be requested from the corresponding author.
Data collection	Data was collected from February to November 2023 at Leiden University Medical Center in Leiden, the Netherlands.
Outcomes	Safety was assessed as the incidence of adverse events as frequencies with percentages (risk). Protective efficacy was defined as he number of participants that did not develop malaria after controlled human malaira infection (CHMI). Time-to-parasitaemia was defined as the number of days between CHMI and a positive qPCR (cut-off 100 parasites/mL whole blood).

Flow Cytometry

Plots

Confirm that:

☒ The axis labels state the marker and fluorochrome used (e.g. CD4-FITC).

☒ The axis scales are clearly visible. Include numbers along axes only for bottom left plot of group (a 'group' is an analysis of identical markers).

☒ All plots are contour plots with outliers or pseudocolor plots.

☒ A numerical value for number of cells or percentage (with statistics) is provided.

Methodology

Sample preparation	Peripheral blood mononuclear cell isolation using ficol gradient.
Instrument	Cytek Aurora Spectral Flow Cytometer
Software	FlowJo version 10.8.2.
Cell population abundance	All cells were acquired without enrichment. Gating based on cell type specific markers was performed.
Gating strategy	Analysed cell populations were gated as indicated in the Extended Data Fig. 2 and 3.

☒ Tick this box to confirm that a figure exemplifying the gating strategy is provided in the Supplementary Information.
